# Are Applied Growth Factors Able to Mimic the Positive Effects of Mesenchymal Stem Cells on the Regeneration of Meniscus in the Avascular Zone?

**DOI:** 10.1155/2014/537686

**Published:** 2014-08-31

**Authors:** Johannes Zellner, Christian Dirk Taeger, Markus Schaffer, J. Camilo Roldan, Markus Loibl, Michael B. Mueller, Arne Berner, Werner Krutsch, Michaela K. I. Huber, Richard Kujat, Michael Nerlich, Peter Angele

**Affiliations:** ^1^Department of Trauma Surgery, University Medical Center Regensburg, Franz Josef Strauß Allee 11, 93042 Regensburg, Germany; ^2^Department of Plastic and Hand Surgery, University Hospital of Erlangen, Friedrich-Alexander University Erlangen-Nürnberg, 91054 Erlangen, Germany; ^3^Sporthopaedicum Regensburg, 93053 Regensburg, Germany

## Abstract

Meniscal lesions in the avascular zone are still a problem in traumatology. Tissue Engineering approaches with mesenchymal stem cells (MSCs) showed successful regeneration of meniscal defects in the avascular zone. However, in daily clinical practice, a single stage regenerative treatment would be preferable for meniscus injuries. In particular, clinically applicable bioactive substances or isolated growth factors like platelet-rich plasma (PRP) or bone morphogenic protein 7 (BMP7) are in the focus of interest. In this study, the effects of PRP and BMP7 on the regeneration of avascular meniscal defects were evaluated. In vitro analysis showed that PRP secretes multiple growth factors over a period of 8 days. BMP7 enhances the collagen II deposition in an aggregate culture model of MSCs. However applied to meniscal defects PRP or BMP7 in combination with a hyaluronan collagen composite matrix failed to significantly improve meniscus healing in the avascular zone in a rabbit model after 3 months. Further information of the repair mechanism at the defect site is needed to develop special release systems or carriers for the appropriate application of growth factors to support biological augmentation of meniscus regeneration.

## 1. Introduction

Meniscal lesions in the avascular zone are still an unsolved problem. Due to the poor self-healing potential of meniscal tissue in the inner zone, partial meniscectomy often is the only treatment option. However, the meniscus plays an important role in the biomechanics of the knee joint concerning force transmission, shock absorption, provision of joint, stability, lubrication, and proprioception [1]. Consecutively, the loss of meniscus predisposes the knee joint to degenerative changes [2].

Regeneration of meniscus in the avascular zone is possible. In particular, the use of mesenchymal stem cells (MSCs) in a Tissue Engineering approach showed improved healing of meniscal lesions in the avascular zone in animal trials [3–5].

However, in these models, the application of MSCs required a two-step procedure with cell expansion between two operations. In a hypothetical clinical use, such an approach would have high regulatory burdens and costs. Additionally, it is still unclear how MSCs promote healing in a Tissue Engineering approach. Besides the possibility that MSCs serve as the repair cells themselves, it seems more than likely that they promote regeneration by delivery of bioactive substances like growth factors [6].

Platelet-rich plasma (PRP) is a clinically available source for the application of growth factors [7]. Depending on the different ways of preparation, PRP provides a huge variety of multiple growth factors [8]. In clinical use, PRP already showed promising results for the regeneration of different tissue types like rotator cuff [9] and cartilage [10, 11] and for enhanced healing during ligament reconstruction [12]. Positive effects on meniscal healing also seem to be possible.

Additionally isolated growth factors have shown implications for healing of musculoskeletal tissue. Regarding cartilage tissue, BMP7 showed improved proliferation of human chondrocytes [13] and chondrogenic differentiation of adipose tissue derived MSCs [14]. In a phase I clinical study, it showed no dose depending toxicity when injected into osteoarthritic knees [15]. In clinical application, BMP7 revealed improved healing of osteochondral defects of the knee by development of hyaline cartilage-like tissue [16], which is also present in the central avascular part of the meniscus.

The goal of this study was the analysis of a combination of growth factors administered by PRP or of a single growth factor with chondrogenic potential like BMP7 to mimic the role of MSCs for promotion of meniscal healing in the avascular zone. The implication of this one-step biological augmentation on the repair capacity of meniscal tissue should be evaluated. We hypothesized that PRP or BMP7 delivered to meniscal lesions in the avascular zone with a hyaluronan collagen composite matrix are able to improve regeneration in standardized previously described [4, 5] animal models.

## 2. Materials and Methods

To ensure a lasting effect of growth factors directly at the meniscal lesion sites, we decided to deliver PRP or BMP7 with a hyaluronan collagen composite matrix. This scaffold showed positive characteristics as a carrier for biological augmentation in previous studies [3–5, 17].

### 2.1. Composite Scaffolds

The sponge scaffolds were manufactured from 70% derivatized hyaluronan-ester and 30% gelatin as described previously [17, 18]. The hyaluronan component was obtained from the commercially available product Jaloskin (Fidia Advanced Biopolymers, Abano Terme, Italy), which is manufactured from hyaluronate, highly esterified with benzyl alcohol on the free carboxyl groups of glucuronic acid along the polymer. The gelatin component was hydrolyzed bovine collagen type I (Sigma, Taufkirchen, Germany).

The porous scaffolds were manufactured by the solvent casting, particulate leaching technique, using NaCl with grain size of 250–350 *μ*m as primary porogen. Additionally, the insufflating air which replaced the evaporating solvent generated secondary pores with the size of 50–100 *μ*m. Scaffolds had a diameter of 2.2 mm and a height of 3 mm.

### 2.2. In Vitro PRP Analysis

For in vitro analysis of growth factor release kinetics, hyaluronan collagen composite scaffolds were seeded with prepared human PRP. Because of the required amount of blood and the subsequent potential clinical use, we decided to analyze release kinetics with human PRP. The growth factor matrix composites were cultured over a period of 8 days and the release of PDGF, TGF*β*1, and VEGF was measured over time.

### 2.3. Preparation of Human PRP and Loading of Composite Scaffolds

For the in vitro analysis of PRP, human blood was drawn from 4 volunteers with the approval of the local ethical committee. Clotting was prevented with citrate and ACD-A. 10 mL blood was spun down unrestrained at 200 G for 15 minutes and after removal of the erythrocytes-layer again at 4000 G for 15 minutes. The platelet-rich cell pellet was isolated by removal of the plasma [19].

In pretests, microscopical analysis and thrombocytes/cell counts were performed to assess the quality and composition of the pellets and the concentration of thrombocytes. To assess viability of the isolated thrombocytes, the standard procedure of a life-dead kit (LIVE/DEAD Viability/Cytotoxicity Kit (L-3224), Mo Bi Tec, Göttingen) had to be modified as thrombocytes lack sufficient quantity of DNA or RNA to detect dead cells. Therefore, vital cells were stained with calcein AM and a photo was taken under the fluorescence microscope. Another picture of the same section was taken under transmitted light in order to count the total number of cells. Both pictures were put together and transparence reduced to 50% each. The number of vital cells was subtracted from all cells to get the number of dead cells.

For further analysis, hyaluronan collagen composite matrices were seeded with PRP by soaking the buffy coat into the scaffolds.

### 2.4. In Vitro Analysis of Growth Factor Release Kinetics

Four PRP hyaluronan collagen composite matrix constructs of each of the 4 volunteers were cultured in vitro over a period of 8 days in 1 mL autologous plasma.

Concentrations of the growth factors PDGF, TGF*β*1, and VEGF were measured by ELISA technique at 0 h, 8 h, 12 h, 24 h, 48 h, and 192 h (8 days) using kits from R&D Systems: Human PDGF-AB DuoSet (DY222), Human TGF*β*1 DuoSet (DY240), and Human VEGF DuoSet (DY293B). Results of cultured empty control scaffolds were subtracted from the growth factor concentrations obtained from the cultured PRP loaded scaffolds in order to exclude an influence of the remaining small growth factor activity in the autologous plasma.

### 2.5. In Vitro BMP7 Analysis

The effect of BMP7 on chondrogenesis was tested to evaluate the potential use of this isolated growth factor for regeneration in the cartilaginous avascular part of the meniscus. For this analysis, the aggregate culture chondrogenesis model with MSCs of rabbits described by Johnstone et al. [20, 21] was used. After preparation of the MSCs, the pellets were cultured in vitro in chondrogenic medium with different concentrations of BMP7. Chondrogenesis was measured by a collagen II ELISA.

### 2.6. Bone Marrow Harvest and Culture

The bone marrow harvest and cell isolation of MSCs were performed as described elsewhere [20]. Marrow derived cells were harvested from the iliac crest of New Zealand White Rabbits and collected into a heparinized syringe. Dulbecco's modified Eagle's medium (DMEM), low glucose concentration, with 10% fetal bovine serum, 1% penicillin, and 1% Hepes was added to the aspirate. Nucleated cells (20 × 10^6^) were plated in 75 cm^2^ culture dishes and cultivated at 37°C. The medium was changed twice a week until the adherent cells reached 80% confluence.

### 2.7. In Vitro Chondrogenic Differentiation

In vitro chondrogenesis was performed according to recently published protocols [17, 20]. Expanded MSCs were trypsinized, and aggregates of 2 × 10^5^ cells were formed through centrifugation at 2000 RPM for 5 minutes in V-bottomed 96-well plates. Chondrogenic differentiation was induced by treatment with serum-free high-glucose DMEM (Gibco, Invitrogen) containing 100 nM dexamethasone (Sigma, Steinheim, Germany), 1% ITS_3 (insulin-transferrin-selenium solution) (Sigma), 200 *μ*M L-ascorbic acid 2-phosphate (Sigma), 1 mM sodium pyruvate (Gibco Invitrogen), and 10 ng/mL human TGF*β*1 (R&D Systems, Wiesbaden, Germany). Culture time was 21 days.

For analysis of the influence of BMP7 on the chondrogenesis of MSCs of rabbits, 5, 10, 50, 100, or 200 ng/mL BMP7 (generous gift from Genera Biotech, Zagreb, Croatia) was added with or without 10 ng/mL TGF*β*1 to the culture medium.

### 2.8. Collagen II ELISA Analysis for Chondrogenic Differentiated MSC Aggregates

An enzyme-linked immunosorbent assay test for collagen II was performed on chondrogenically differentiated MSC aggregates. Pellets were homogenized in 0.05 M acetic acid plus 0.5 M NaCl (pH 2.9-3.0), digested with 10 mg/mL pepsin dissolved in 0.05 M acetic acid on the rotator for 48 hours at 4°C. The further steps of digestion and the collagen type II estimation were performed as described in the Native Type II Collagen Detection Kit 6009 protocol (Chondrex, Redmond, WA, USA). The DNA concentration in collagen digests was assayed using the Quant-iT PicoGreen dsDNA Assay Kit (Invitrogen, Eugene, OR, USA). Collagen type II was determined as a ratio between content of Collagen type II and DNA for each pellet.

### 2.9. In Vivo Analysis of the Effects of Applied PRP or BMP7 on Meniscal Lesions in the Avascular Zone

Harvest of platelet-rich plasma and loading of composite scaffolds for the animal trial: for the animal trail, autologous blood (10 mL) was drawn from the anesthetized rabbit's ear vein. This procedure was approved by the Local Institution of Animal Care. The preparation of the PRP and the seeding of the scaffolds were done according to the human protocol described above.

### 2.10. Surgical Procedure for Meniscus Defects

The rabbit animal models were already described and are validated standardized models for testing of meniscal treatment in the avascular zone [3–5]. Similar to human meniscus untreated or only sutured lesions in the avascular zone show no tendency for healing. The procedures were approved by the Institutional Animal Care and Use Committee of our institution.

24 New Zealand White rabbits (five-month-old males) were used for the in vivo PRP analysis. The rabbits were anesthetized and exposure of the lateral joint compartment was achieved by a lateral parapatellar arthrotomy. Avascular meniscal defects were made by using a 2 mm punch device (Stiefel, Offenbach am Main, Germany) (12 rabbits) or by inserting a 4 mm long longitudinal meniscal tear in the avascular zone (12 rabbits). The punch defects were treated with a hyaluronan collagen composite matrix loaded with PRP. The meniscal tears were treated by a PRP seeded composite matrix and a 5–0 PDS outside-in suture. This procedure was done bilaterally, with the contralateral knee serving as control; an empty hyaluronan-gelatin scaffold was the control implant for all rabbits. Postoperatively, the animals were allowed free movement without use of any type of immobilization. Rabbits started full weight bearing immediately after recovery from anesthesia. The animals were sacrificed at 6 or 12 weeks. Each group consisted of six New Zealand White rabbits.

For the in vivo evaluation of BMP7 effects on meniscal healing, 12 animals were used. A 2 mm circular shaped meniscal defect in the avascular zone was inserted and treated with a hyaluronan collagen composite matrix and an additional injection of 1 *μ*g BMP7 at the time of implantation (Group 1, 6 rabbits). In another group, the defect was filled with a 14-day precultured construct of MSCs and a hyaluronan collagen composite matrix (Group 2, 6 rabbits). Harvesting of the MSCs and seeding of the scaffold was performed like described above [5]. Each scaffold was seeded with 1.5 × 10^6^ MSCs. The chondrogenic medium consisted of DMEM (high glucose), 200 *μ*M ascorbic acid 2-phosphate, 1% ITS (both from Sigma, Taufkirchen, Germany), 1 mM pyruvate, 100 nM dexamethasone, 10 ng/mL TGF*β*1 (R&D systems, Wiesbaden, Germany), and 50 ng/mL BMP7. The implantation of a cell-free hyaluronan collagen composite matrix in a 2 mm circular avascular defect in the lateral meniscus of the contralateral side served as a control group. Follow-up period was 3 months.

### 2.11. Gross Assessment of Joint Morphology

Rabbits with surgical implants were euthanized for tissue harvest with an overdose of pentobarbital (1600 mg/mL) given intraperitoneally. After exposure of the knee joint, the macroscopic morphology of the meniscus and the attachments of the meniscus to the tibial plateau were evaluated and photographed.

### 2.12. Histology

The lateral menisci harvested from the in vivo experiments were fixed in 4% phosphate buffered paraformaldehyde embedded in Tissue-Tek O.C.T. and frozen in liquid nitrogen. Ten-micrometer radial sections of all samples were produced and every fifth of them was stained with toluidine blue or DMMB.

### 2.13. Immunohistochemistry

As the pars intermedia of rabbit's meniscus contains mainly collagen type II, especially towards the avascular central part of the meniscus, the immunohistochemical analysis was performed for collagen type II. Sections were washed and then digested for 15 min with 0.1% pepsin at pH 3.5 to facilitate antibody access to the target epitopes. Type II collagen was immunolocalized by the immunoperoxidase ABC technique (Vector, Burlingame, CA, USA), applying monoclonal primary antibodies ms. anti collagen II, clone II-4C11 (Calbiochem-Merck, Schwalbach, Germany), biotin conjugated polyclonal secondary antibodies (goat anti-mouse IgG (Jackson, West Grove, PA, USA)), and the nickel and cobalt enhanced DAB stain visualization.

### 2.14. Meniscus Scoring System

In order to compare the macroscopical, histological, and immunohistochemical results after repair of the meniscal lesions, a validated meniscus scoring system was used, which was developed and published for the evaluation of meniscal defects [4, 5]. Subgroups in macroscopical assessment were “stability” and “defect filling with repair tissue” and for histological analysis the “quality of the surface area,” “integration,” “cellularity,” and “cell morphology” and subgroup for immunohistochemical characterization was the “expression of proteoglycan and moderate collagen type II in the repair tissue.” The repair was graded by summing up the scores from 0 to 3 of eight individual subgroups. Consequently, the final scores were between 0 points (no repair) and maximal 24 points (complete reconstitution of the meniscus) (Table 1). The data was collected from 2 blinded scorers, both experienced in knee anatomy of rabbits and in histological assessment.

### 2.15. Statistical Analysis

For the in vitro BMP7 evaluation, independent unpaired *t*-tests were performed to compare the different collagen II ELISA groups. For the in vivo testing, the scoring results of each group were compared to the results of the control group (cell-free hyaluronan collagen composite matrix on the contralateral side). Paired *t*-tests were done for the analysis of the scoring results of all groups. For all evaluations, the level of statistical significance was set at a probability value of less than 0.05.

## 3. Results

### 3.1. In Vitro Analysis of PRP

Human PRP seeded in hyaluronan collagen composite matrices resulted in a high number of vital thrombocytes (94%). The PRP was leukocyte-poor with an average of 2,5 × 10^7^ platelets/*μ*L and a 3 times higher concentration of thrombocytes compared to the corresponding blood samples. After seeding of the composite matrix, an equal distribution of the thrombocytes throughout the scaffold was obtained (data not shown).

**Figure 1:**
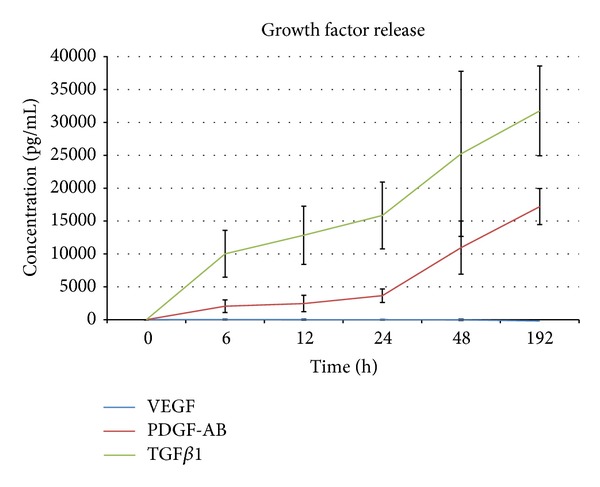
Release kinetics of the growth factors TGF*β*1, PDGF, and VEGF from PRP hyaluronan collagen composite matrix constructs over a period of 8 days cultured in rabbits' autologous plasma (mean values of 4 volunteers with standard deviation).

To imitate the joint environment, the PRP/hyaluronan collagen composite matrix constructs were cultured for 8 days in autologous plasma. The results of the ELISA analysis showed a constant increase in PDGF and TGF*β*1 from day 0 to day 8 indicating that growth factors were released over the whole follow-up period. No VEGF was detectable over the period of 8 days (Figure 1).

### 3.2. In Vivo Analysis of the Meniscal Treatment in the Avascular Zone with PRP

**Figure 2:**
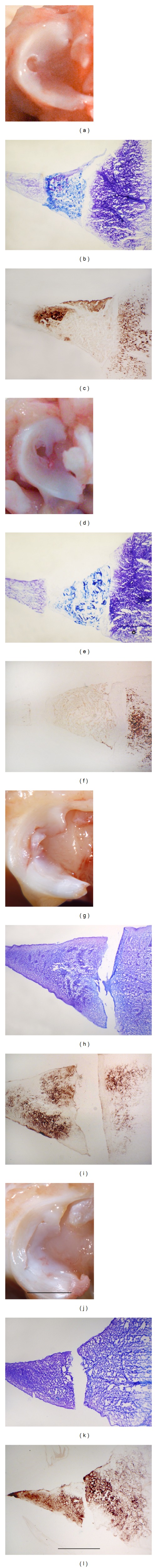
Gross morphology and histological and immunohistochemical (collagen type II) analysis 3 months after treatment of a meniscal punch defect with a PRP hyaluronan collagen composite matrix construct (a, b, and c) or an empty matrix (control) (d, e, and f) and 3 months after treatment of meniscal tears with a PRP hyaluronan collagen composite matrix construct (g, h, and i) or an empty matrix (j, k, and l). No improvement by treatment of meniscal lesions with PRP could be detected. Magnification bars: (a, d, g, and j): 10 mm; (b, c, e, f, h, i, k, and l): 1 mm.

The implantation of a hyaluronan collagen composite matrix loaded with PRP showed no significant improvement of the repair of avascular meniscal punch defects compared to an implantation of a cell-free scaffold. After 6 and 12 weeks, the lesions were only partially filled with fibrous-like scar tissue. Tears in the tip of the native meniscus could often be detected (Figures 2(a), 2(b), and 2(c)).

**Figure 3:**
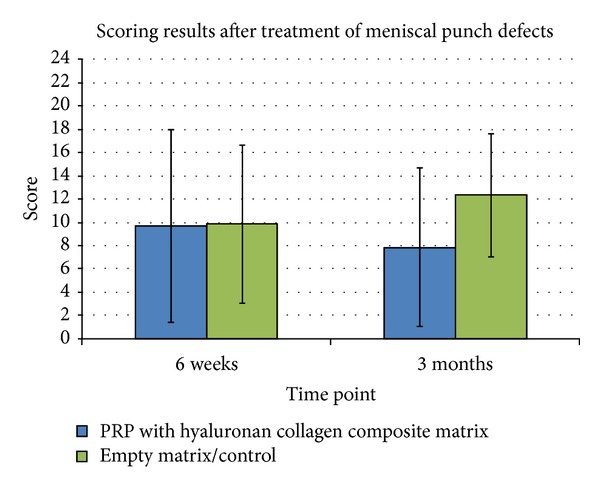
Results of the scoring of meniscal repair tissue after 6 weeks and 3 months in vivo. No significant improvement by treatment of a meniscal punch defect with PRP could be detected compared to the control group (*P ≤ 0,05).

In the control group, repair of the punch defects with cell-free matrices resulted in partial defect filling in half of the animals after 6 weeks and also after 12 weeks (Figures 2(d), 2(e), and 2(f)). Macroscopically, the repair tissue was soft and only partially integrated. Microscopically, the punch defects were partially filled with fibrous and cell-rich scar tissue. No residuals of the implanted scaffolds could be detected (Figure 3).

**Figure 4:**
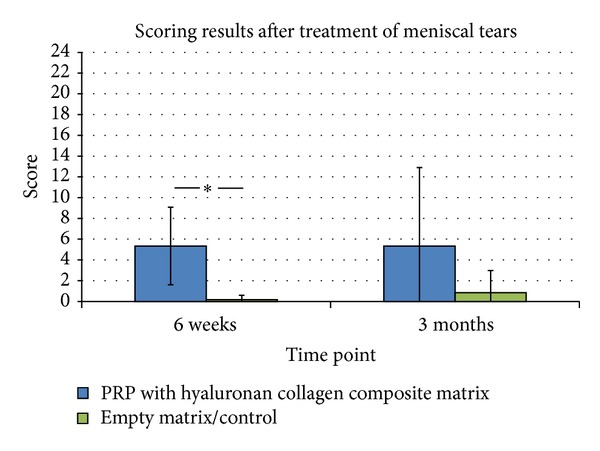
Results of the scoring of meniscal repair tissue after 6 weeks and 3 months in vivo. Significant improvement by treatment of meniscus tears with PRP could be detected after 6 weeks compared to the control group (*P ≤ 0,05).

Regarding the meniscus tear model, a significant better repair of avascular meniscal tears could be detected after treatment with PRP seeded matrices compared to the cell-free matrices after 6 weeks (*P* < 0,05). However, this positive effect of PRP was not significant after 3 months mainly due to a high inter-animal variability. Defect filling with constructs containing matrices with PRP resulted in a poor tear filling without regeneration of the meniscal tear after 3 months. In a few cases, muted instable fibrous attachments between the two parts of the meniscus could be detected (Figures 2(g), 2(h), and 2(i)). No signs of meniscus-like tissue reconstitution could be seen (Figures 2(j), 2(k), and 2(l)). In contrast to complete empty tears in the control group, this mutant repair tissue was responsible for the improved scores (Figure 4).

### 3.3. In Vitro Analysis of BMP7

All tested BMP7 concentrations, added to chondrogenic medium with TGF*β*1, revealed chondrogenic differentiation of MSCs. The addition of 50 ng/mL BMP7 showed the best results regarding chondrogenesis in the pellet culture model with the highest content of collagen II in the ELISA analysis. The addition of higher concentrations of BMP7 showed no beneficial effect on the development of collagen II under TGF*β*1 medium condition.

**Figure 5:**
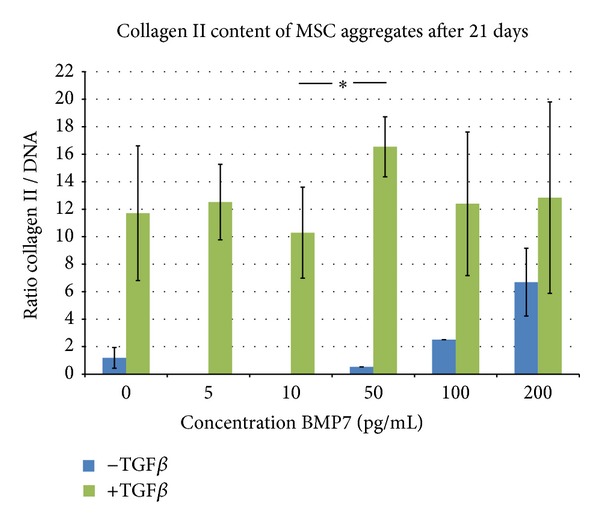
ELISA analysis of the collagen II content of chondrogenic differentiated mesenchymal stem cell (MSC) aggregates after 21 days of culture under different conditions. The pellets were cultured with 0, 5, 10, 50, 100, or 200 ng/mL BMP7 with or without 10 ng/mL TGF*β*1. The addition of 50 ng/mL BMP7 to the TGF*β*1 containing culture medium showed the highest content of collagen II compared to other BMP7 concentrations with a significant difference between 10 ng/mL and 50 ng/mL BMP7 (*P ≤ 0,05). A BMP7 concentration dependent increase in collagen II content was detected under culture conditions without TGF*β*1.

In culture condition without TGF*β*1, BMP7 showed a concentration dependent increase in collagen II deposition but less chondrogenic differentiation compared to TGF*β*1 containing conditions (Figure 5).

### 3.4. In Vivo Analysis of the Influence of BMP7 on the Regeneration of Meniscal Defects

**Figure 6:**
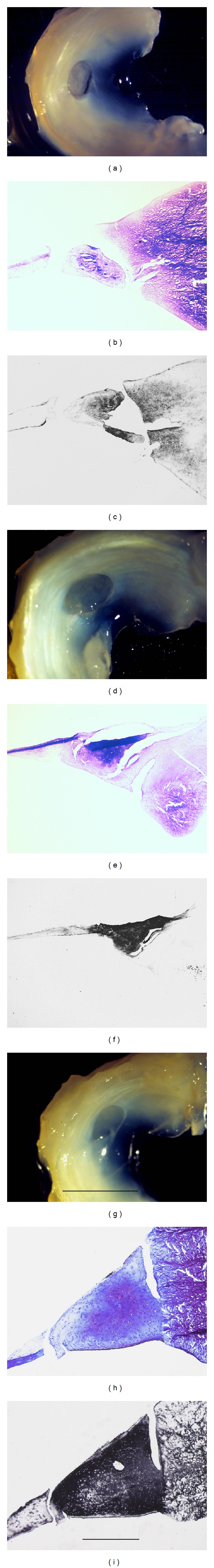
Gross morphology and histological and immunohistochemical (collagen type II) analysis 3 months after treatment of a meniscal punch defect with a cell-free hyaluronan collagen composite matrix and a single injection of 1 *μ*g BMP7 at the time of surgery (a, b, and c) or with an empty matrix (control) (d, e, and f). Images (g, h, and i) show the results after treatment with a hyaluronan collagen composite matrix seeded with mesenchymal stem cells (MSCs) and precultured in chondrogenic medium containing BMP7 for 14 days. Treatment with MSCs showed the best defect filling with differentiated repair tissue. Magnification bars: (a, d, and g): 10 mm; (b, c, e, f, h, and i): 1 mm.

**Figure 7:**
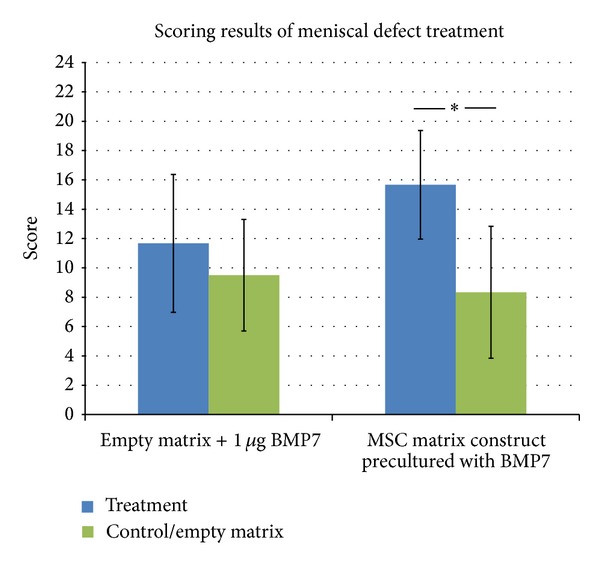
Results of the scoring of meniscal repair tissue after 3 months in vivo. Treatment with mesenchymal stem cell composite matrix constructs showed significant repair improvement compared to the control group (*P ≤ 0,05).

The additional injection of 1 *μ*g BMP7 in meniscus lesions at the time of treatment of a circular avascular meniscal defect with cell-free hyaluronan collagen composite matrices (group 1) showed no beneficial effect compared to matrix implantation without BMP7 injection (control). After 3 months in vivo, only mixed tissue with scar and small-differentiated areas (collagen type II positive) were detectable in the BMP7 treated meniscal defects and in the control defects (Figures 6(a)–6(f)). However, the defects treated with MSC composite matrix constructs and precultured in a BMP7 and TGF*β*1 containing chondrogenic medium showed superior meniscal scoring results compared to the cell-free matrices (Figure 7). In defects treated with precultured MSC matrix constructs, differentiated meniscus-like repair tissue was detectable after 3 months in vivo. In contrast, the treatment with a cell-free composite matrix showed only fibrous defect filling after 3 months in vivo (Figures 6(g)–6(i)).

## 4. Discussion

The study analyzed the effects of PRP on meniscus regeneration in two different meniscus defect models. PRP seeded hyaluronan collagen composite matrices failed to repair a circular full size meniscal defect as well as meniscus tears in the avascular zone. After 3 months, the local injection of BMP7 in composite matrices for treatment of circular meniscal defects in the avascular zone showed no improvement of meniscus regeneration compared to treatment with composite matrices without BMP7. Only treatment with constructs of autologous MSCs seeded on a hyaluronan collagen composite matrix showed improvement of meniscal healing and defect filling with differentiated meniscus-like tissue after 3 months in vivo. Nevertheless, growth factors are still in the focus of a potential clinical use for biological augmentation of meniscus treatment as they provide the possibility of a one-step procedure.

Tissue Engineering is a promising therapy option for the treatment of meniscal lesions especially in the avascular zone. Recent studies showed that MSCs are able to fill avascular meniscal defects with differentiated repair tissue [3–5]. However, these approaches require a two-step procedure with the need of cell expansion between two operations. Such approaches would have high regulatory burdens and costs in daily clinical practice.

Additionally, it is still unclear how MSCs promote meniscal healing. Caplan and Dennis [6] described a dual role of MSCs in musculoskeletal regeneration. On the one hand, MSCs could differentiate into repair cells that are required at the defect site. On the other hand, MSCs could act as a mediator for bioactive substances and secrete, for example, growth factors. So it seems very likely that the use of growth factors only could have similar positive effects on the regeneration of meniscus tissue compared to a stem cell based approach by mimicking the delivery of bioactive substances.

PRP represents an easy available source for a combination of multiple growth factors that is already in clinical use and can be applied in a one-step procedure. Properties like “biological glue,” contribution to coagulation and hemostasis, intra-articular restoration of hyaluronic acid, anti-inflammation, and pain relief are described [7].

Beneficial effects by clinical use of PRP were seen in treatment of rotator cuff tears [9], Achilles tendon ruptures [22], chronic tendinosis [23], muscle injuries [7], ACL-rupture [12], and cartilage defects [11, 24].

Different techniques were described to prepare PRP. The quality and composition of the PRP depend on the speed and number of centrifugations, the use of anticoagulant or activator, and the presence of leukocytes [7]. The PRP used in this study was leukocyte-poor so that a described deleterious effect from matrix metalloproteinases 8 and 9 from the neutrophils in the PRP [25] could be neglected. The number of platelets in the PRP is essential for their biological potential. For treatment of bone defects, defined concentrations of platelets in the PRP are described for optimal effects on bony regeneration [26]. However, there are no data in literature that evaluated the most effective concentration of platelets and released growth factors for a biological support of meniscus regeneration. Additionally, in this study, the way of preparation of the PRP had to be adapted to the rabbit model. In order to reach a high number of active and vital thrombocytes, decision was made for an unrestrained centrifugation with 200 G for 15 minutes and 4000 G for another 15 minutes with ACD-A and citrate to inhibit coagulation. By this method, a high number of vital thrombocytes were reached with only 10 mL blood of the rabbits.

Growth factor release was measured over a period of 8 days. In order to imitate the synovial fluid environment of the knee, the PRP hyaluronan collagen composite matrix constructs were cultured in rabbits' autologous plasma. Constant release of PDGF and TGF*β*1 that are known to enhance differentiation and proliferation of meniscal cells [27, 28] was seen over the whole measure period of 8 days. The content of collagen type I in the composite matrix might be a possible reason for the constant release of growth factors, as collagen type I is known as an activator for PRP, for example, from chitosan matrices [29]. Similar to this study, Harrison et al. saw a constant prolonged release of growth factors compared to other activators like thrombin when collagen type I was used as a component of a PRP seeded scaffold [30].

However, no release of VEGF was detectable over 8 days. While other authors report a high concentration of VEGF in the PRP [8], recently, Anitua et al. also saw a fast decrease in VEGF release from their PRP matrix [31]. The different methods of preparation or presence of soluble VEGF receptors from remaining leukocytes [31] might be possible reasons for the varying amounts of VEGF. Theoretically, a highly angiogenic growth factor like VEGF [32] might have a positive effect on the regeneration of an avascular tissue like the inner zone of the meniscus. However, there are reports that VEGF coated PDLLA sutures failed and showed even worse results than uncoated sutures when meniscal tears in the avascular zone of meniscus were reconstructed in a rabbit model [33]. So VEGF does not seem to be a mandatory factor for regeneration in the avascular zone of meniscus.

In this study, PRP delivered to an avascular meniscal defect in combination with a hyaluronan collagen composite matrix failed to improve meniscal healing. No sufficient repair tissue was detectable in the circular punch defect after 6 or 12 weeks. However, Ishida et al. showed positive results in vitro and in vivo by treatment of avascular meniscal defects with PRP [34], but the meniscal defect size was smaller than that in the present study.

In treatment of meniscus tears, a tendency of improved healing with the addition of PRP to the meniscal suture could be seen after 6 weeks; however, this effect was not significant after 3 months in vivo mainly due to a high inter-animal variability. Partially stable repair tissue was detectable with the addition of PRP, which was responsible for the higher scores compared to complete empty tears in groups with meniscus suture alone. Clinically, Kessler and Sgaglione [35] explored the clinical use of PRP to augment meniscal repairs and found successful healing along with an 80% success rate in Tegner and Lysholm scores of 40 young patients treated with meniscal repair and PRP. However, this clinical study was a case series without a control group. So there is still no clear evidence of improvement of meniscal healing with PRP, but signs for a positive influence on meniscal regeneration.

Besides the application of a combination of multiple growth factors with PRP, also isolated growth factors are interesting for enhancement of meniscal repair in a clinical one-step setting. One of these growth factors that are clinically applicable is BMP7. BMP7 showed promising results for induction of bone formation [36] but also in the field of cartilage therapy. So BMP7 improved the culture and proliferation of human chondrocytes [13] and enhanced the chondrogenic differentiation of adipose tissue derived MSCs in vitro. Cook et al. were able to successfully treat osteochondral defects with BMP7 injection in clinical use [16]. In this study, the addition of BMP7 to the chondrogenic medium with TGF*β*1 induced higher contents of collagen II in chondrogenically differentiated aggregates of MSCs. However, high concentrations of BMP7 in culture conditions without TGF*β*1 showed also increasing contents of collagen II deposition indicating the highly chondrogenic potential of this growth factor.

In vivo, the local injection of BMP7 at the defect site in addition to the insertion of a hyaluronan collagen composite matrix showed partially differentiated repair tissue but no significant improvement of meniscal healing in an avascular meniscal punch defect compared to a matrix without BMP7. In contrast, treatment of meniscal punch defects with a MSC composite matrix construct resulted in a significant improvement of meniscal healing in the avascular zone.

In this study, BMP7 was added to the chondrogenic medium during the 14 days of preculturing period of the MSC composite matrix constructs. As comparable results in treatment of avascular meniscal defects were achieved without the use of BMP7, in recent studies, BMP7 does not seem to be mandatory in the preculturing period.

Limitations of the study are the rabbit animal model and the different cell sources used in the study that make the results less comparable.

PRP and BMP7 failed to significantly improve meniscal healing in vivo in this animal model. Nevertheless, short term improvement in treatment of meniscal tears by PRP, constant release of growth factors from a PRP seeded hyaluronan collagen composite matrix, and support of MSCs by BMP7 are promising aspects for a possible clinical application of growth factors to support meniscal treatment. As a promising biological augmentation applicable in a one-step procedure, growth factors still have to be in the focus of future research. One of the actual problems for treatment with bioactive substances like PRP or isolated growth factors might be the uncontrolled manner of acting at the defect site. As MSCs promote meniscal healing, their secretion pattern of bioactive substances has to be elucidated to be able to apply the right growth factors with the correct concentration at the right time during the regeneration process. Specific release systems and carriers will be necessary to reach that goal.

## 5. Conclusions

In the current study, PRP and BMP7 showed positive aspects to promote meniscus regeneration in a one-step procedure but failed to improve significantly meniscal healing in the avascular zone in vivo. Uncontrolled release of growth factors in vivo might be a possible reason. However, biological augmentation for regenerative meniscal treatment in a one-step procedure still seems to be possible. One aspect of further investigations might be the analysis of the effective secretion patterns of bioactive substances of MSCs to develop release systems for a defined and specific application of growth factors at the meniscal defect site.

## Figures and Tables

**Table 1:** Scoring system for the evaluation of the quality of meniscal repair tissue.

	0	1	2	3
Defect filling	No fill	<25%	25–75%	>75%

Surface	No surface	ruptured	Fissured/fibrillated	Meniscus-like

Integration	No integration	Partial, unilateral integration	Bilateral partial or unilateral complete integration	Bilateral complete integration

Cellularity	No cells	>10 cell clusters/slide	No cell cluster/slide, cell-ECM-ratio >0,5	Meniscus-like cell-ECM-ratio

Cell morphology	No cells	<25% meniscus-like cells	25–75% meniscus-like cells	>75% meniscus-like cells

Content of proteoglycan	No staining for proteoglycan	<25%	25–75%	>75%

Content of collagen II	No staining for collagen II	<25%	25–75%	>75%

Stability	No stability	Weak	Stable in shape	Stable to pressure and pulling stress

ECM: extracellular matrix.
